# Will exposure to different consequences of prosocial behavior always lead to subsequent prosocial behavior among adolescents: An experimental study of short videos

**DOI:** 10.3389/fpsyg.2022.927952

**Published:** 2022-09-29

**Authors:** Wu Li, Yuanyi Mao, Bo Hu

**Affiliations:** ^1^School of Media and Communication, Shanghai Jiao Tong University, Shanghai, China; ^2^Department of Media and Communication, City University of Hong Kong, Hong Kong, Hong Kong SAR, China

**Keywords:** prosocial media content exposure, prosocial behavior, moral elevation, empathy, late adolescents

## Abstract

The relationship between exposure to prosocial media content and prosocial behavior has been extensively explored. However, previous studies mainly explore the effect of prosocial media content exposure by comparing an individual’s exposure to the different types of content (i.e., prosocial content or neutral content), and generally focus on traditional media and video games, with less attention given to the increasingly popular new media platforms. In this study, we explored new dimensions by considering individuals’ exposure to different consequences of the same prosocial behavior (i.e., reward, punishment, or no consequences) in the context of short videos. Drawing upon Social Cognitive Theory and the General Learning Model, this experimental study identified the effect of such exposure on subsequent prosocial behavior among adolescents. We found that compared to the no consequences group, exposure to the reward consequence did not significantly predict moral elevation and subsequent prosocial behavior. Meanwhile, exposure to the punishment consequence had a significantly negative effect on subsequent prosocial behavior *via* moral elevation. Furthermore, the results revealed that empathy moderated the relationship between moral elevation and prosocial behavior, and moral elevation only positively predicted prosocial behavior among those with low empathy. Theoretically, this study deepens our understanding of the impact of exposure to different consequences of prosocial behavior on adolescents’ subsequent prosocial behavior, and highlights the importance of moral elevation and empathy to understand the underlying mechanism. The study also provides some practical implications for parents and practitioners to nurture prosocial behavior among adolescents.

## Introduction

Prosocial behavior is a voluntary and intentional behavior resulting in others’ benefits (Eisenberg and Miller, 1987). Research on this topic originated in psychology with McDougall (1908), who argued that prosocial behavior was the result of “tender emotions” created by the parental instinct, and has burgeoned since Darley and Latane (1968)’s scientific inquiry into the non-responsive bystanders in the brutal murder of Katherine “Kitty” Genovese in 1964. Recent research shows that performing prosocial behavior is not only helpful for others, but also beneficial for actors themselves, particularly for adolescents (Penner et al., 2005; Aknin et al., 2018). For instance, adolescents’ prosocial behavior is proved to be positively associated with their academic performance (Gerbino et al., 2018), friendship quality (Closson, 2009), well-being (Son and Padilla-Walker, 2020), and achievement at later life stages (Toumbourou, 2016). Moreover, given that adolescence is the stage when one’s values and worldviews are formed, it is often recognized as a key period for prosocial development (Foulkes et al., 2018). Therefore, scholars have explored the predictors of prosocial behavior to better nurture and advance adolescents’ prosocial behavior (Eisenberg, 2003; Carlo et al., 2011; Imuta et al., 2016; Li et al., 2022).

In the field of media study, considerable research endeavors have been devoted to establishing the relationship between exposure to prosocial media content and individuals’ prosocial behavior in the past several decades (Saleem et al., 2012; Greitemeyer and Mügge, 2014; Prot et al., 2014; Mares and Stephenson, 2017; Ruth, 2017; Coyne et al., 2018). For instance, a study found that children who were exposed to prosocial television news donated more money to charities compared to those who watched neutral news (de Leeuw et al., 2015). Likewise, exposure to Disney animation movies in which the main character helped friends effectively facilitated children’s prosocial behavior toward their friends in real life (de Leeuw and van der Laan, 2018). A laboratory experiment on music consumption revealed a similar result that participants’ empathy and prosocial behavior significantly increased after they listened to music with prosocial lyrics (Greitemeyer, 2009a,b). In general, the positive effect of consuming prosocial media content has been repeatedly confirmed with few exceptions (Coyne and Padilla-Walker, 2015; Padilla-Walker et al., 2015).

The issue with most of the previous studies, however, is that they explored the effect of prosocial media content on prosocial behavior by comparing an individual’s exposure to different types of content, such as prosocial content vs. neutral content. In fact, prosocial content delivered in the media is much more complicated and defies simple categorization. For instance, people are sometimes exposed to media content that depicts performance of a certain prosocial behavior with different outcomes: positive or negative. A positive outcome may come in the form of receiving a verbal compliment, an honorary title, or a material reward, while a negative outcome in the form of being misunderstood, blackmailed, or even being at the risk of an arrest (Smith et al., 2006). This kind of media content, such as the sensational Peng Yu case in 2007^1^ (Wang et al., 2019) and similar events that occurred more recently in China, reflects what often happens in our society, i.e., “good things happen to good people” or “no good deed goes unpunished.” Considering the prevalence of such media content, research at a more nuanced level needs to be conducted by taking into consideration individuals’ exposure to different consequences of the same prosocial behavior.

In addition, a review of the literature on prosocial media content exposure and prosocial behavior yielded another observation. Most extant studies were conducted in such traditional media contexts as television (de Leeuw et al., 2015; Padilla-Walker et al., 2015), movies (de Leeuw and van der Laan, 2018), and music (Greitemeyer, 2009b). Despite the extension into video games (Gentile et al., 2009; Greitemeyer and Osswald, 2009; Greitemeyer and Mügge, 2014; Prot et al., 2014), little attention has been given to new media such as the increasingly popular short videos. Short videos are considered short in length, from several seconds to several minutes depending on the platform (Wang and Wu, 2021). Because of their short length, they are more easily shared on today’s social media compared to television programs and movies. In fact, short videos have already gained rapid growth and attracted millions of users worldwide (Omar and Dequan, 2020). According to industry reports, the number of daily active users on TikTok, one of the most attractive short video platforms, has already reached 400 million in China (Marszałek, 2020) and 800 million worldwide (DataReportal, 2022). Among these active users, young people aged between 16 and 24 are the predominant users (Beer, 2019), and their average daily time spent on TikTok is 45 min (Holmes, 2019). Meanwhile, due to the bite-sized duration, short videos often need to present the content in a more vivid and even dramatic way to attract people’s attention compared to traditional media (Peng, 2018). The consumption of short videos thus may elicit users’ strong cognitive and emotional responses (Li et al., 2020), which have been proven to be the important processes underlying people’s prosocial decision-making (Rahal and Fiedler, 2022). Therefore, it is necessary to go beyond the contexts of traditional media and video games to explore the effect of consuming prosocial media content in the increasingly popular short video arena.

In sum, to better understand the relationship between prosocial media content exposure and subsequent social behavior, we intend to explore new dimensions by examining individuals’ exposure to different consequences of the same prosocial behavior (i.e., reward, punishment, or no consequences) in the context of short videos. Late adolescents are sampled because they are in a crucial developmental period of worldview exploration marked by instability and uncertainty (Arnett, 2000; McLean, 2005) and thus are easily susceptible to external influences. Meanwhile, late adolescents are old enough to self-report a measurable impact on their moral change, and actual behavior. Drawing on Social Cognitive Theory (SCT) and the General Learning Model (GLM), we built a moderated mediation model with moral elevation as a mediator and empathy as a moderator, and then conducted data analyses to test the proposed hypotheses.

## Theoretical background and hypothesis development

### The effects of exposure to different consequences of prosocial behavior

According to the SCT, people can learn behaviors vicariously by observing other people’s actions and the ensuing consequences (Bandura, 1986). This observational learning process can occur in person or from media displays. Due to the individual’s limited time, resources, and biological restrictions, people cannot acquire all their knowledge and behaviors directly from personal experiences. Instead, most people’s attitudes, values, and behavioral patterns are shaped by what they observe in their media environment (Bandura, 2001). However, it is important to emphasize that although people might acquire certain behaviors from role models in mediated environments, they will not perform all the learned behaviors in real life.

To a great extent, observationally learned behaviors depend on vicarious motivations (Bandura, 2001), which mainly stem from the consequences of role models’ behavior. Specifically, when the observed character gains reward outcomes for his/her behavior, observers may be incentivized to perform a similar behavior. In contrast, when the character receives punishments for his/her behavior, it could discourage observers from imitating the displayed actions (Bandura, 1969, 2001, 2004). The classic experiment based on Social Learning Theory is Bandura’s (1965) Bobo Doll study. This early experiment demonstrates that different outcomes of a behavior can have varying influences on observers’ adoption of such behavior, although it focused on aggressive behavior rather than prosocial behavior.

In today’s media environment, people are likely to be repeatedly exposed to media characters experiencing different consequences for their actions, which in turn might elicit their cognition and behavioral change (Mayrhofer and Matthes, 2020). For example, Mayrhofer and Naderer (2019) found that the portrayal of positive consequences of consuming alcohol in movies or TV dramas increases positive expectations and attitudes about alcohol among those with low alcohol consumption. In the domain of media and moral behaviors, there also existed a few studies investigating how exposure to media characters experiencing different consequences for their actions influence individuals’ moral-related behaviors. For example, Lee et al. (2014) found that listening to “George Washington” stories, which emphasized the positive consequences of being honest, would increase children’s truth-telling behaviors. Similarly, Yao and Enright (2020) randomly assigned kindergarten children to listen to either a moral story with good consequences or a control story with no consequences, and they found children in the reward group shared more candies with other kids compared with those in the control group. These studies show that observing characters behaving altruistically with good consequences can effectively promote an observer’s execution of prosocial behavior. Based on the results of these studies, we can also argue that exposure to the punishment consequence of prosocial behavior will discourage people from imitating the same prosocial behavior to a great extent.

In short, based on the rationale of the SCT and previous empirical studies, we hypothesize as follows:

H1: Individuals exposed to the reward consequence of prosocial behavior demonstrate more subsequent prosocial behavior.

H2: Individuals exposed to the punishment consequence of prosocial behavior demonstrate less subsequent prosocial behavior.

### The mediating role of moral elevation

The GLM is informed by the SCT and related social-cognitive research. This framework posits that situational (e.g., media exposure) and individual factors jointly influence a person’s cognition, feelings, and physiological arousal, which affect their ensuing behaviors (Buckley and Anderson, 2006). Studies have demonstrated that exposure to prosocial media successfully activated individuals’ accessibility of prosocial thoughts (Greitemeyer, 2011). However, compared to studies on cognitive change (such as moral judgment and moral reasoning) triggered by viewing prosocial media content (Eisenberg, 1986; Walker, 2004; Carlo, 2006), the research on emotional responses to such content has been seldom examined. In fact, when people are watching prosocial or morally virtuous video clips, their moral-related emotions, as indicated by the GLM, might be activated. More research thus needs to be done to understand the mechanism behind such responses.

Moral elevation is a moral emotion that can be potentially induced when people witness others’ virtuous acts or prosocial behavior (Ding et al., 2018). As a multi-dimensional construct, elevation emotion consists of several components such as thoughts, feeling, motivation, and physiological changes. For example, after seeing moral behavior in others, observers may experience a sense of warmth and pleasantness, have uplifted and inspired feeling, possess optimistic thoughts about humanity, desire to be a better person, and emulate the observed moral behavior (Pohling and Diessner, 2016; Thomson and Siegel, 2017). In the field of media psychology, evidence from several empirical studies suggests that people’s moral elevation will increase when they are exposed to prosocial content in video clips (Oliver et al., 2012, 2015; Lai et al., 2014; Krämer et al., 2021).

However, the change of moral elevation could be more complicated if individuals are exposed to different consequences of prosocial media content. In addition to the experience of moral elevation triggered by the observed prosocial behavior itself, the reward consequence may also induce feelings of appreciation and admiration. A study by Pohling and Diessner (2016) revealed that the state of moral elevation is an emotion which could be strengthened by admiration and appreciation. Consequently, it is reasonable to argue that observers may experience a higher level of moral elevation when they find that the media character receives the reward outcome after engaging in a certain prosocial behavior, compared to those exposed to prosocial media content with no consequences.

By contrast, when people see someone doing a good deed yet receiving punishment, they will judge such an outcome as injustice because it violates the moral standard of fairness (Graham et al., 2013). Numerous studies have shown that observing such unethical outcomes happened to another person can trigger witnesses’ moral outrage, a mixed feeling of anger and disgust (Wakslak et al., 2007; Salerno and Peter-Hagene, 2013; Antonetti and Maklan, 2016). Conceptually, moral outrage has opposing emotional valence toward moral elevation; thus, the increased moral anger will inhibit the feeling of moral elevation. Moreover, the feeling and expressions of moral outrage are much more easily amplified by digital media due to its technological affordances (Crockett, 2017). Therefore, we argue that observers may experience a lower level of moral elevation when they find the media character in short videos gets punished after performing a certain prosocial behavior.

H3a: Compared to exposure to prosocial behavior with no consequences, exposure to the reward consequence has a positive relationship with individuals’ moral elevation.

H3b: Compared to exposure to prosocial behavior with no consequences, exposure to the punishment consequence has a negative relationship with individuals’ moral elevation.

At the same time, people who experience moral elevation have a strong motivation and tendency to emulate moral exemplars and behave in a prosocial manner (Haidt, 2003). Moral elevation has been consistently found to be a significant predictor of people’s prosocial behavior. For instance, Schnall et al. (2010) found that participants experiencing moral elevation spent much more time helping experimenters with tedious tasks than those in the control group. Freeman et al. (2009) showed that the experience of moral elevation led people to donate more money to charitable organizations. Other studies also indicated that individuals who have experienced moral elevation are more likely to offer help and develop more life goals related to morality (Algoe and Haidt, 2009; Van de Vyver and Abrams, 2015). Thus, we propose the following hypotheses:

H4: Moral elevation positively predicts subsequent prosocial behavior.

In sum, based on the theoretical assumptions and extant literature, we argue that compared to exposure to prosocial behavior with no consequences, exposure to reward and punishment consequence predicts individuals’ moral elevation positively and negatively. And then we expect a positive relationship between moral elevation and subsequent prosocial behavior. Given these conceptual arguments, the variable of exposure to different consequences of prosocial behavior, moral elevation, and subsequent prosocial behavior are linked. Therefore, we hypothesize:

H5a: Moral elevation mediates the relationship between exposure to the reward consequence and subsequent prosocial behavior.

H5b: Moral elevation mediates the relationship between exposure to the punishment consequence and subsequent prosocial behavior.

### The moderating role of empathy

As the GLM suggests, personal factors interact with media exposure to impact people’s decision-making. Empathy is one such factor, and its role is especially important when it comes to individuals’ altruistic behavior. Defined as an “other-oriented emotion elicited by and congruent with the perceived welfare of someone in need” (Batson, 2011, p. 2), empathy includes tenderness, sympathy, and compassion (Batson, 2011). According to the empathy-altruism hypothesis, Batson (2011) argued that individuals’ empathy is associated with their altruistic motivation and prosocial behavior. People who are in low levels of empathy are usually more aggressive and suffer interpersonal problems (Vachon et al., 2014; Mitsopoulou and Giovazolias, 2015). Meanwhile, moral elevation seems to be an effective way of mitigating the detrimental effects of low empathy since it is effective in instigating subsequent prosocial actions (Haidt, 2003). Therefore, we are curious about the conjoined role of moral elevation and empathy in promoting prosocial behavior.

The scarcity of published literature on this issue also suggests a need to further understand the interplay between moral elevation and empathy. Although no study directly examined the relationship between these two constructs, a few pieces of literature addressed the interactive effects of moral emotions and empathy-related concepts on prosocial behaviors, which could provide insights for our study. For instance, Zuffianò et al. (2015) examined the effect of the interrelationship between respect for moral others (a positive emotion which is similar to moral elevation in our study) and sympathy (a concept similar to empathy in that both imply caring for another person albeit minute differences) in promoting children’s sharing behavior. They found that respect for moral others was positively associated with sharing behavior only among children who were in the low sympathy group. Oriol et al. (2020) investigated the interactive effect of self-transcendent aspiration (a concept close to moral elevation) and empathy on gratitude (an important predictor for prosocial behaviors), and found that the effect of self-transcendent aspiration on gratitude was stronger for people with low empathy than those with high empathy. These studies suggest that moral elevation may play a compensatory role in facilitating prosocial behavior for people with low empathy. Based on previous studies, we argue that moral elevation serves as a compensatory function to some extent in promoting prosocial behavior for individuals with a low level of empathy.

H6: Empathy moderates the influence of moral elevation on prosocial behavior, and moral elevation has a greater positive effect on subsequent prosocial behavior in people with low empathy compared to those with high empathy.

**FIGURE 1:**
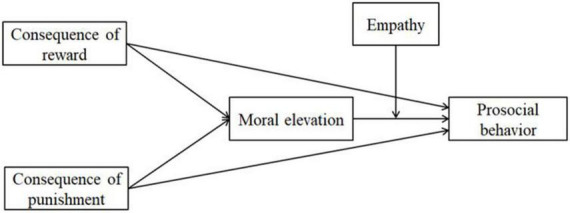
Conceptual model.

Based on the above hypotheses, the conceptual model for this research was depicted in Figure 1.

## Materials and methods

### Participants

Experimental research was adopted for this study, considering it is the best way to infer causality (Bazaraa et al., 2022). Specifically, we chose a between-group design to collect data from the target population which in our study is late adolescents, young people about 17–19 years old as defined by Eisenberg et al. (1995). There were two reasons for us to focus on late adolescents. First, adolescents are in an important phase of prosocial development and they are also susceptible to external influences, thus deserving more academic attention. Second, compared to those in early or middle adolescence, those in late adolescence are old enough to self-report a measurable impact on their moral change and actual behavior.

We recruited senior high school students and college freshmen from a middle school and a university located in Shanghai, China. Before recruiting participants, we used G*Power to calculate the minimum sample size. The results showed that 121 participants are needed to achieve a medium effect size of 0.15 and a minimum power of 0.8 (Faul et al., 2007) in multiple regression with ten predictors (two independent variables, one mediator, one moderator, one interaction term, and five covariates). In total, 124 students participated in our laboratory experiment. Among them, the mean age is 17.960 (*SD* = 1.393), 47.6% are female, 57.3% are the single child in their family, and the majority (80.6%) reported that they had no religious beliefs. Despite the non-probability sampling for data collection, the sample distribution basically matched the profile of Chinese adolescents, particularly in terms of gender and religious beliefs (Gao, 2016; Office of the Leading Group of the State Council for the Seventh National Population Census, 2022).

### Procedures

The study adhered to the tenets of the Declaration of Helsinki, and all of the procedures were approved by the authors’ Institutional Review Board (No. H2021177I). All participants were invited to our college laboratory one by one and were told that the purpose of our experiment was to examine how watching short videos influences their cognitive abilities. To minimize the potential harm caused by environmental and other factors, we invited the participants to the same physical laboratory to participate in the experiment, and the research procedures were carried out consistently from the first participant to the last one.

First, they filled out the consent form and completed a pre-test questionnaire on demographic information and empathy. Then, they were randomly assigned to the different groups, namely, different consequences of prosocial behavior: getting a reward (*N* = 43), getting a punishment (*N* = 41), or no consequences (*N* = 40). To ensure that participants carefully watched these video clips, they were informed to take a memory test related to the content in the video afterward.

After viewing a version of the short videos, participants were asked to answer manipulation check questions and moral elevation measures. They were also required to write down the amount they would like to donate from their incentive money after reading a hypothetical charitable request. Next, we checked whether the participants had any suspicion regarding the relationship between watching short videos and the donation task, and found no one suspected the purpose of our experiment. Finally, we debriefed the participants, explaining the real objective of our study, and thanked them for their participation with 20 RMB cash.

### Stimulus

In order to find appropriate experiment stimulus for this study, we first searched for relevant short videos on TikTok, using keywords such as “helping” and “good deed.” This resulted in hundreds of clips whose content included but was not limited to strangers’ helping elderly people and drivers’ returning wallets to the owner. Following a rigorous selection process, we chose three short videos from the search results. The criteria for selection were 3-fold. The content must contain clear references to typical prosocial behavior, the images should be clear enough to discern, and the subtitles need to be easy for re-editing.

After that, one professional video-editor was recruited to edit the three short videos. Three versions for each short video were created, each corresponding to one of the three conditions in our study: the reward condition, the punishment condition, and the control condition. In order to maximally reduce the potential impacts of other factors, all the elements of the short video were kept the same except for the subtitles appearing on the screen at the end of the video. Finally, all three versions from the same condition were put into the same group: the reward group, the punishment group, and the control group (the details of the stimulus material provided in the Appendix).

#### Pilot testing of videos

To guarantee the effectiveness of these short videos, we conducted a pilot test. We anticipated that participants could discern the different consequences of prosocial behavior portrayed in the video clips while maintaining the same evaluation of other dimensions (i.e., objectiveness, credibility, relevance, and amusement of the short videos) across different groups.

We recruited 30 participants and randomly assigned them to the reward and punishment conditions. The participants were then instructed to answer to what extent they agree that (1) the helpers in the short videos got a reward, and (2) the helpers in the short videos got a punishment. Participants were asked to rate on five-point scales ranging from one (“strongly disagree”) to five (“strongly agree”). Also, they were asked to evaluate the objectiveness, credibility, relevance, and amusement of the short videos. As we expected, compared to those in the punishment group (*M* = 2.444, SD = 1.247), participants in the reward group (*M* = 4.250, SD = 0.754) reported significantly higher scores that the helpers got reward [*t*_(28)_ = −4.484, *p* < 0.001]. Likewise, compared to those in the reward group (*M* = 1.333, SD = 0.492), participants in the punishment group (*M* = 3.111, SD = 1.183) scored significantly higher on the punishment question [*t*_(28)_ = 4.909, *p* < 0.001]. No significant differences were found between the two groups in their assessment of the objectiveness, credibility, relevance, and amusement of the short videos they watched. Thus, the stimulus was appropriate to be used in our experiment.

### Measures

#### Pre-experimental measures

Demographics: Participants were required to report their gender, age, religious belief, and whether they are the single child in their families. Past research suggested that these demographic variables were significant predictors of prosocial behavior (Bekkers and Wiepking, 2011; Wiepking and Bekkers, 2012; Watanabe and Lee, 2016). Thus, these factors were included as covariates in our study.

Empathy: Given the important role of empathy, especially the emotional empathy, in predicting adolescents’ prosocial behavior (Zhang et al., 2021), empathy was measured by the dimension of empathic concern in the Interpersonal Reactivity Index (Davis, 1983). We adopted the seven items and participants rated the extent to which they disagree or agree on a five-point scale (1 = strongly disagree, and 5 = strongly agree). Example items included “I am often quite touched by things that I see happen,” “I would describe myself as a pretty soft-hearted person,” and “When I see people being taken advantage of, I feel kind of protective toward them.” All the items were averaged to indicate the degree of empathy, and a higher score indicates a higher level of empathy. We examined the reliability and validity of this scale by using confirmatory factor analyses, and the results showed that it had good internal reliability and construct validity: χ^2^/df = 1.723, *p* = 0.026, IFI = 0.967, CFI = 0.966, GFI = 0.941, RMSEA = 0.077, and SRMR = 0.063 (Cronbach’s α = 0.701).

#### Post-experimental measures

Manipulation check questions: Participants were asked to rate on a five-point Likert scale (1 = strongly disagree, 5 = strongly agree) to what extent they agree that (1) the helpers in the short videos got a reward and (2) the helpers in the short videos got a punishment.

Moral elevation: Eight items were adopted from the study of Aquino et al. (2011) which measured participants’ view of humanity and their desire to become a better person after seeing prosocial actions in the short videos. Examples include “There is still some good in the world,” “The world is full of kindness and generosity,” “The actions of most people are admirable.” Participants were asked to rate these items using a five-point Likert scale ranging from one (“strongly disagree”) to five (“strongly agree”). All items were averaged to indicate the degree of their moral elevation. We conducted confirmatory factor analyses of the scale and it was demonstrated to have good internal reliability and construct validity: χ^2^/df = 1.390, *p* = 0.148, IFI = 0.952, CFI = 0.949, GFI = 0.956, RMSEA = 0.056, and SRMR = 0.054 (Cronbach’s α = 0.820).

Prosocial behavior: In the present study, prosocial behavior was represented by the participants’ donation behavior. Participants were exposed to a donation request for a hypothetical charitable project. Describing the life struggles of the old who suffer from cataracts, the project claimed to raise money to help cure the elderly. At the end of the request, participants were instructed as follow: “If you decide to donate a portion of your payment to this project, we will pay that amount directly to the charity, and compensate you with the remaining amount” (Thomson and Siegel, 2017, p. 54). Then, participants were asked how much money they would like to donate (ranging from 0–20 RMB). The monetary amount they chose to donate was measured as their prosocial behavior (*M* = 8.826, SD = 7.062).

### Data analyses

Before testing our hypotheses, we made the no consequences group (control group) the reference group. Thus, consequence of reward (i.e., reward vs. no consequences) and consequence of punishment (i.e., punishment vs. no consequences) were created as independent variables. PROCESS macro for SPSS was adopted to test the hypotheses, and demographic variables were entered into each model as covariates.

## Results

### Manipulation check and descriptive statistics

In our study, we found that participants in the reward group (*M* = 4.581, SD = 0.698) reported that the helpers in the short videos received significantly more rewards than those in the control group [*M* = 3.000, SD = 1.396, *t*_(81)_ = 6.597, *p* < 0.001]. Also, compared to the control group (*M* = 1.275, SD = 0.599), participants in the punishment condition (*M* = 3.488, SD = 1.247) scored significantly higher in response to the question to what extent they agreed the helpers in the short videos received a punishment [*t*_(79)_ = −10.216, *p* < 0.001]. These results indicated we successfully manipulated the consequences of helpers’ prosocial behavior in the video clips.

Additionally, we also found that participant’s gender [χ^2^_(2)_ = 0.659, *p* = 0.719], monthly disposable income [χ^2^_(10)_ = 14.935, *p* = 0.134], single-child status [χ^2^_(2)_ = 5.614, *p* = 0.060], religious belief [χ^2^_(2)_ = 3.346, *p* = 0.188] are independent across the different experimental conditions. The ANOVA test [*F*_(2,121)_ = 6.082, *p* = 0.003] and *post hoc* comparisons revealed that participants’ age was higher in the control group (*M* = 18.500, SD = 0.934) compared to the punishment group (*M* = 17.463, SD = 1.733), and there was no significant difference in age between the reward group (*M* = 17.930, SD = 1.223) and the punishment group (*p* = 0.113). In addition, The ANOVA test also showed that participants’ empathy was independent form the different experimental conditions [*F*_(2,121)_ = 0.809, *p* = 0.448]. These results suggested that these factors are basically equally distributed across the experimental conditions.

The descriptive statistics of moral elevation, empathy and prosocial behavior in each condition were presented in Table 1.

**TABLE 1:** Descriptive statistics.

Variables	Experimental conditions	Mean [95% CI]	SD
Moral elevation	Control group	3.888 [3.719, 4.056]	0.528
	Reward group	3.971 [3.826,4.116]	0.470
	Punishment group	3.402 [3.195,3.610]	0.657
Empathy	Control group	3.571 [3.409, 3.734]	0.509
	Reward group	3.728 [3.548, 3.908]	0.585
	Punishment group	3.631 [3.442, 3.819]	0.598
Prosocial behavior	Control group	9.275 [7.194, 11.356]	6.508
	Reward group	9.430 [7.203, 11.657]	7.236
	Punishment group	7.754 [5.407, 10.100]	7.435

### Moderated mediation analysis

To test our hypotheses, we conducted a moderated mediation analysis using PROCESS Macro for SPSS model 14 (Hayes, 2017), in which the group was entered as the independent variable, prosocial behavior as the dependent variable, moral elevation as the mediator, and empathy as the moderator. Since the independent variable of the group was a nominal variable with three categories, we dummied it with the control condition as the baseline group, resulting in two specific independent variables: X1 (the reward group) and X2 (the punishment group). In addition, following the suggestions of Rosenthal and Cummings (2021) on considering covariates in addition to experimental effects in data analysis, we controlled demographic factors (e.g., gender, age, single child status, religious belief, and monthly disposable income) in the model examination to better understand the impact of different consequences of prosocial behavior on participants’ moral elevation and subsequent prosocial behavior.

**FIGURE 2:**
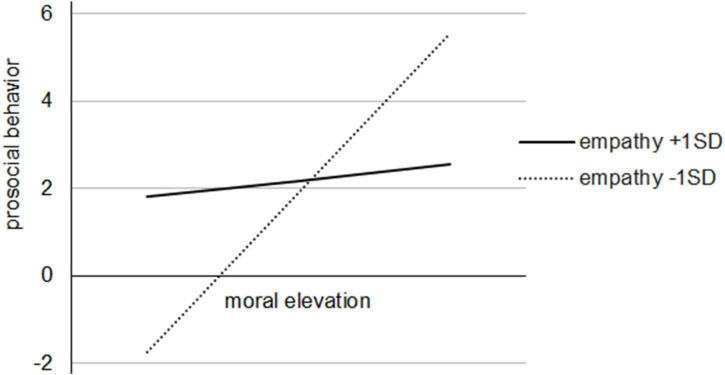
Plot of interaction of moral elevation and empathy on prosocial behavior.

We first examined the regression analyses output of the two models with moral elevation and prosocial behavior as dependent variables respectively (see Table 2). Model 1 showed that compared to the control group, the punishment consequence significantly predicted participants’ moral elevation (Coeff. = −0.517, *p* < 0.001), whereas the effect of the reward consequence on moral elevation was not significant (Coeff. = 0.080, *p* = 0.533). In Model 2, the results showed that neither the reward consequence (Coeff. = 0.961, *p* = 0.531) nor the punishment consequence (Coeff. = 1.206, *p* = 0.481) significantly predicted prosocial behavior compared to the control group. Moral elevation (Coeff. = 3.306, *p* = 0.008) significantly predicted prosocial behavior, whereas, empathy did not (Coeff. = 0.244, *p* = 0.835). Furthermore, the interactive effect of moral elevation and empathy was significant (Coeff. = −4.745, *p* = 0.017), supporting the moderating effect of empathy on the relationship between moral elevation and prosocial behavior. To better understand this interactive effect, we conducted a simple slope test and plotted the relationship when empathy was below and above one standard deviation of the mean. As can be seen in Figure 2, for adolescents with low empathy, moral elevation positively predicted prosocial behavior (effect = 5.987, *p* = 0.001); however, this effect was not significant for those with high empathy (effect = 0.624, *p* = 0.667).

**TABLE 2:** Testing of the moderated mediation model.

	Consequent					
	Model 1 (moral elevation)			Model 2 (prosocial behavior)		
Antecedents	Coeff.	SE	*p*	Coeff.	SE	*p*
X1 (Reward group)	0.080	0.127	0.533	0.961	1.528	0.531
X2 (Punishment group)	−0.517	0.132	<0.001	1.206	1.707	0.481
Mediator (Moral elevation)	−	−	−	3.306	1.216	0.008
Moderator (Empathy)	−	−	−	0.244	1.168	0.835
Moral elevation × Empathy	−	−	−	−4.745	1.963	0.017
Constant	1.115	1.020	0.276	−2.037	12.324	0.869
**Covariates**						
Gender^a^	0.071	0.103	<0.001	1.826	1.223	0.141
Age	−0.073	0.052	0.162	0.349	0.632	0.582
Single children^b^	0.090	0.114	0.433	0.440	1.379	0.750
Religious beliefs^c^	−0.020	0.149	0.894	−0.417	1.780	0.815
Monthly disposable income	0.060	0.050	0.232	0.738	0.608	0.228
	*R*^2^ = 0.196			*R*^2^ = 0.184		
	*F*_(7,116)_ = 4.048, *p* < 0.001			*F*_(10,113)_ = 2.547, *p* = 0.008		

^a^Male = 0, female = 1. ^b^Yes = 0, no = 1. ^c^Yes = 0, no = 1.

To test the conditional indirect effects, we then employed the bootstrap confidence interval recommended by Preacher and Hayes (2004). If a confidence interval for the indirect effect does not straddle zero, it can statistically support that M mediates the effect of X on Y at that value of the moderator (Hayes and Rockwood, 2017). As is displayed in Table 3, a 95% bootstrap confident interval based on 5,000 bootstrap samples indicated Path 1 (the reward consequence → moral elevation → prosocial behavior) was not contingent upon empathy level, since the 95% bootstrap confident interval of the index of moderated mediation straddled zero (−1.812, 0.887). In contrast, Path 2 (the punishment consequence → moral elevation → prosocial behavior) was contingent upon empathy level since the corresponding CI value was entirely above zero (0.468, 5.672). To be specific, among those with low empathy, the specific indirect effect of the punishment consequence on prosocial behavior through moral elevation was significant (effect size = −3.094, BootCI: [−5.859, −1.114]), whereas among those with high empathy, the specific indirect effect was non-significant (effect size = −0.322, BootCI: [−1.777, 1.424]).

**TABLE 3:** Indices of moderated mediation with 95% bootstrap confidence intervals.

					Empathy							
	Index of moderated mediation				Low				High			
Path	Index	Boot SE	Boot LLCI	Boot LLCI	Effect	Boot SE	Boot LLCI	Boot ULCI	Effect	Boot SE	Boot LLCI	Boot ULCI
Path 1	−0.377	0.650	−1.812	0.887	0.476	0.748	−1.038	2.052	0.050	0.224	−0.400	0.564
Path 2	2.452	1.323	0.468	5.672	−3.094	1.215	−5.859	−1.114	−0.322	0.790	−1.777	1.424

Path 1: the reward consequence → moral elevation → prosocial behavior; Path 2: the punishment consequence → moral elevation → prosocial behavior.

## Discussion

Drawing upon the SCT and the GLM, this experimental study examined the effects of exposure to different consequences of prosocial behavior on adolescents’ subsequent prosocial behavior in the context of short videos. The study found that compared to the no consequences group, exposure to the reward consequence did not significantly predict moral elevation and subsequent prosocial behavior. Meanwhile, exposure to the punishment consequence had a significantly negative effect on subsequent prosocial behavior *via* moral elevation. Furthermore, the results revealed that empathy moderated the relationship between moral elevation and prosocial behavior and moral elevation only positively predicted prosocial behavior among those with low empathy. More discussion of the key findings is presented as follows.

Surprisingly, contrary to our expectation, the results did not reveal any significant effects of the reward consequence stimulus on observers’ moral elevation or their prosocial behavior. The discrepancy between this finding and our expectation based on the SCT (Bandura, 2001) and previous empirical studies (e.g., Lee et al., 2014; Yao and Enright, 2020) could be possibly explained by the long-term effects of moral education in China. Chinese students normally receive moral education based on the national curricula since an early age for nearly 10 years in school settings (Cheng, 2019). The textbooks in the curriculum of moral education present various moral exemplars, aiming at encouraging children to emulate their prosocial behavior (Han et al., 2018). Such long-term moral education might lead to two social-psychological consequences.

One consequence is the desensitization of prosocial media content, which refers to a decrease in cognitive or emotional responses to repeated exposure to moral-related media content (Krahé et al., 2011). Thus, when the participants in our research were exposed to the reward consequence of prosocial behavior, their emotional arousal would be difficult to trigger. Consequently, no evidence of the influence of reward stimulus on moral elevation or prosocial behavior was found. The other possible consequence is the process of social norm internalization (Cheng, 2019). After receiving long-term moral education, students will gradually internalize societal norms (such as the social responsibility to help others in need) into their personal beliefs, and might think that doing good deeds is inspired by their intrinsic motivations rather than external rewards and incentives. Studies have shown that prosocial behavior motivated by personal norms is independent of external environments stimulus (van der Linden, 2011). Therefore, the participants in our experiment would have little or no behavioral responses when they witnessed prosocial behavior with external reward incentives.

The story of the punishment condition is somewhat different from the reward condition. Specifically, the study revealed that compared to the control condition with no consequences, the punishment stimulus had a negative effect on participants’ moral elevation and subsequent prosocial behavior. The different effects of the punishment/reward consequences of prosocial behavior on observers are interesting, and the possible explanation might be a “negativity bias.” As a kind of cognitive bias, negativity bias refers to people’s propensity to engage in quick autonomous cognitive processing and pay more attention to negative information than positive information (Rozin and Royzman, 2001). More importantly, negative events tend to elicit more prominent and stronger emotional responses in people than positive events (Carretié et al., 2009). Thus, the participants in our study were easily aroused by the punishment stimulus and experienced stronger moral emotions compared to those exposed to the reward stimulus.

Another finding worth discussing is the moderation effect of empathy, which provides us with an insightful look into the interrelated effect of moral elevation and empathy on ensuing prosocial behavior. In line with Zuffianò et al.’s (2015) study, we found that there was a positive relationship between moral elevation and prosocial behavior in adolescents with low empathy, yet such a relationship diminished among those with high empathy. This result indicates that moral elevation serves as a compensatory function to some extent in promoting prosocial behavior for individuals with a low level of empathy. In other words, the influence of the punishment consequence on adolescents’ subsequent prosocial behavior *via* moral elevation was contingent on the level of empathy, which helped us understand the underlying mechanism at a more nuanced level.

## Implications and limitations

Our study has made several theoretical contributions. First, it yields a more nuanced view concerning the influence of media content exposure on adolescents’ subsequent prosocial behavior by focusing on the different consequences of the same prosocial behavior depicted in the media. Most of the previous studies explored the effect of prosocial media exposure by comparing an individual’s exposure to the different types of content, such as prosocial content vs. neutral content. Our study took a different approach to address this issue. We distinguished between reward and punishment consequences from the same prosocial behavior, and examined people’s altruistic outcomes after being exposed to either consequence. Second, to the best of the authors’ knowledge, our research study represents the first attempt to investigate such an important topic in the context of short videos. Considering its uniqueness and increasing popularity among people’s life, our study enriches and extends the current knowledge on the effect of prosocial media exposure by going beyond the traditional media context on which most of the extant studies focus. Third, by introducing moral elevation as the mediator and empathy as the moderator into the proposed model, our study uncovered the psychological mechanism which shed light on adolescents’ prosocial learning process in the context of the new media environment, namely, how and when exposure to the different consequences of prosocial behavior influences their subsequent prosocial behavior. Lastly, our findings lend support to the application of the SCT and the GLM in this study, which in turn provide new evidence to support the explanatory power of these two theories in a new context.

Our study also has some implications for practice. Considering that exposure to the punishment consequence of prosocial behavior will decrease young viewers’ moral elevation and prosocial behavior, measures could be taken by parents and practitioners (such as school teachers) to develop their morals and behavior in a more prosocial way. For example, adolescents should be protected from frequent exposure to short videos containing the consequential punishment, and psychological intervention is needed to moderate the negative outcomes when they are found to have consumed excessive amount of such media content. Also, adolescents should be guided to discern and stand against the actions of “porcelain bumping” depicted in the media content^2^ (Li, 2019), which corresponds to helpers’ getting punished after their performing prosocial behavior in our study. What’s more, keeping the moderating effect of empathy in mind, short video platforms can fully take advantage of big data to discern adolescents with different levels of empathy, and recommend more videos with rewards for good deeds to those with lower empathy in order to nurture their prosocial behavior.

Despite the above contributions, this study suffers several limitations. First, our study analyzed the short-term effects of prosocial media exposure on individuals’ prosocial behavior in adolescents. Longitudinal studies on exposure to different consequences of media prosocial behavior are needed for more insightful results. Second, we used donation behavior to represent adolescents’ prosocial behavior. Since there are other types of prosocial behavior such as helping and sharing (Gross et al., 2015), a more comprehensive measure of prosocial behavior needs to be considered in future research. Third, given the fact that moral elevation is only one facet of the multidimensional construct of moral emotion, it is necessary to consider other moral emotions, such as guilt or disgust, as possible intervening variables to gain a more comprehensive understanding of the underlying mechanism.

## Data availability statement

The raw data supporting the conclusions of this article will be made available by the authors, without undue reservation.

## Ethics statement

The studies involving human participants were reviewed and approved by the Institutional Review Board of Shanghai Jiao Tong University (No. H2021177I). Written informed consent to participate in this study was provided by the participants’ legal guardian/next of kin.

## Author contributions

WL: conceptualization, methodology, data analysis, writing – review and editing, and funding acquisition. YM: methodology, investigation, data analysis, and writing. BH: investigation and data analysis. All authors contributed to the article and approved the submitted version.

## Footnotes

1 Peng Yu case is a civil lawsuit in China brought before the Nanjing District Court in 2007. In 2006, Peng Yu, a young man, encountered an old lady called Xu Shoulan after she had fallen and broken her femur. Peng Yu assisted Xu Shoulan and brought her to a local hospital for care. However, Xu Shoulan accused Peng Yu of having caused her to fall and demanded that he pay her medical expenses. The court decided in favor of the plaintiff, reasoning that despite the lack of concrete evidence, “no one would in good conscience help someone unless they felt guilty.”

2 “Porcelain bumping” is called “Pengci” in Chinese, which refers to a practice in contemporary China whereby scammers feign injury or financial loss in public to extort money from strangers. It origins from the act that crooks deliberately put fragile porcelain in a place where it can easily be bumped in order to claim the compensations.

## Appendix

**Appendix Table 1:** Detailed materials of manipulated story plots for short video stimulus.

	Plot description of video clip	Manipulation in the reward group	Manipulation in the punishment group	Manipulation in the control group
Story 1	The taxi driver Qi Junlan found a wallet in her cab, which contained cash, ID cards, and bank cards. She immediately contacted the owner of the lost wallet.	The owner got the wallet back, and gave some money to the driver as a form of gratitude.	The owner insisted that the driver stole the cash and called the police. Driver Qi felt deeply wronged.	The owner got his wallet back.
Story 2	A female teacher met an old man who had fallen down on the road and drove him to the hospital.	The old man’s family members thanked the teacher and gave her a silk banner as an award for her school.	The old man’s family members accused the female teacher for hitting the old man and asked for medical compensation.	The teacher drove the old man to the hospital and left.
Story 3	A high school student helped an old man who fell from his bike.	The old man contacted the student’s school, and expressed his gratitude to the boy for his good deeds.	The old man contacted the student’s school, and insisted that it was the boy who had knocked him down, and asked for medical compensation.	The student helped the old man up and then left.
